# Outbreak of Zika Virus Infections, Dominica, 2016

**DOI:** 10.3201/eid2311.171140

**Published:** 2017-11

**Authors:** Sadie J. Ryan, Colin J. Carlson, Anna M. Stewart-Ibarra, Mercy J. Borbor-Cordova, Moory M. Romero, Shelly-Ann Cox, Roché Mahon, Adrian Trotman, Sylvester St. Ville, Shalauddin Ahmed

**Affiliations:** University of Florida, Gainesville, Florida, USA (S.J. Ryan); University of KwaZulu-Natal School of Life Sciences, Durban, South Africa (S.J. Ryan);; University of California, Berkeley, California, USA (C.J. Carlson);; State University of New York Upstate Medical University, Syracuse, New York, USA (A.M. Stewart-Ibarra, M.M. Romero);; Escuela Superior Politécnica del Litoral, Guayaquil, Ecuador (M.J. Borbor-Cordova);; Caribbean Institute of Meteorology and Hydrology, Bridgetown, Barbados (S.-A. Cox, R. Mahon, A. Trotman);; Ministry of Health and Environment, Roseau, Commonwealth of Dominica (S. St. Ville, S. Ahmed)

**Keywords:** Zika virus, ZIKV, flavivirus, pandemic, autochthonous, microcephaly, sexual transmission, Commonwealth of Dominica, Brazil, Pan American Health Organization, Caribbean, Puerto Rico, *Aedes*, *aegypti*, *albopictus*, mosquitoes, vector-borne infections, viruses

## Abstract

In February 2016, the World Health Organization declared the pandemic of Zika virus a public health emergency. On March 4, 2016, Dominica reported its first autochthonous Zika virus disease case; subsequently, 1,263 cases were reported. We describe the outbreak through November 2016, when the last known case was reported.

Zika virus is a flavivirus transmitted primarily by *Aedes aegypti* and *Ae. albopictus* mosquitoes. The rapid spread of Zika virus from Brazil throughout the Americas, and the associated emergence of Zika congenital syndrome, which causes microcephaly and other birth defects, has posed an unprecedented challenge to global health (*1*,*2*). Zika virus spread through the Caribbean region early in the pandemic. In epidemiologic week 19 of 2015, Brazil reported its first confirmed locally acquired cases. Autochthonous transmission in Martinique was first reported in epidemiologic week 51 of 2015, and the first case originating in Puerto Rico was reported in week 52 of 2015 (*3*). Many other islands began reporting cases of Zika virus infection early in 2016. However, case data from several Caribbean countries has yet to be consolidated and described outside of reports by the Pan American Health Organization.

We defined suspected Zika virus disease cases in the Commonwealth of Dominica, on the eastern sector of the Caribbean Sea, by using guidelines provided by the Pan American Health Organization (*4*). Active surveillance of cases (suspected and confirmed) among persons who visited health clinics started as early as January 2016; however, the first laboratory-confirmed autochthonous case of Zika virus disease was identified in March 2016. We collected data from records in the Ministry of Health and Environment, Dominica, describing patients’ age, sex, residence, date of illness onset, clinical features, laboratory diagnoses, and travel history.

The first case of laboratory-confirmed Zika virus disease in Dominica was reported on March 4, 2016 (during epidemiologic week 9), in a 28-year-old woman. New cases (suspected and confirmed) were reported that year through November 6 (Figure). The last cases in 2016 were reported in epidemiologic week 44. A total of 1,263 suspected cases of Zika virus disease were reported in Dominica in 2016, of which 79 (6.25%) were confirmed by using reverse transcription PCR. Of these, only 1 specimen tested negative but was classified as a suspected case. 

**Figure:**
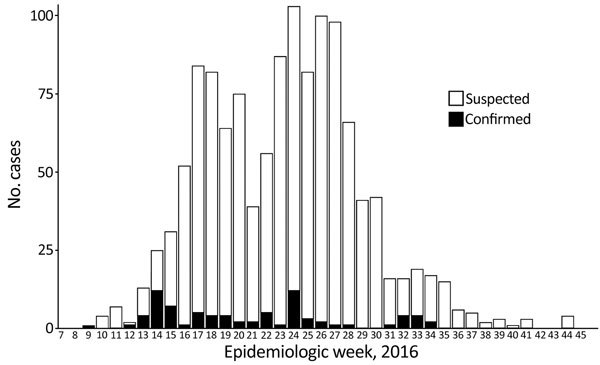
Suspected and confirmed cases of Zika virus infection reported during outbreak, Dominica, 2016.

Sex was reported for 1,255 (99.3%) of 1,263 case-patients. Approximately twice as many case-patients were female (863, 68.8%) than male (392, 31.2%), which is consistent with a female bias found in other reports on Zika virus disease outbreaks (*5*). 

Age was reported for 1,245 (98.6%) of 1,263 case-patients. Mean age was 28 years (median 27, range <1–94 years). Of those, 217 (17.4%) case-patients were children <10 years of age and 756 were of reproductive age (15–49 years); 555 (73.4%) of reproductive age case-patients were women. 

Of the 1,240 case-patients for whom age and sex were reported, 555 (44.8%) were women of childbearing age. Pregnancy status was only reported for 54 (6.3%) of 863 female case-patients; of those, 16 (29.6%) were pregnant. Of the 16 pregnant women, 11 were confirmed case-patients, and disease was suspected in 5.

Of the 1,263 total case-patients, clinic visit dates were recorded for 1,123 (88.9%), and 27 (2.1%) reported hospitalizations. The average number of days between the onset of symptoms and initial clinic visit was 1.96 (n = 1,109; 5%, 95% quantiles = 0, 5); the average number of days between a recorded clinic visit and case reporting was 0.5 (n = 1,096; 5%, 95% quantiles = 0, 10). Of the 27 (15 female/11 male/1 unknown) hospitalizations, 2 were for women reported to be pregnant, and 6 were for children <10 years of age.

*Aedes* spp. mosquitoes are widespread throughout the Caribbean and are associated with Zika, dengue, and now chikungunya viruses, which are endemic to many islands. As part of the broader pandemic in the Americas, the Zika virus disease outbreak in Dominica highlights that the presence of *Aedes* spp. can be predictive of outbreaks. Dominica has a population of ≈72,000, on an island of 750 km^2^, and ≈1.67% of the population were reported to have suspected or confirmed cases of Zika virus disease in 2016. 

We found a bias toward infection in women, including a substantial number of women of childbearing age, highlighting the potential vulnerability of pregnant women and unborn children. As was seen during the recent epidemic of chikungunya in Dominica (*6*), the rapid proliferation of Zika virus disease cases emphasizes the need to strengthen local capacities for targeted vector control and global efforts to support development of effective vaccines, in addition to a better understanding of the role of sexual transmission and the heightened risk to vulnerable populations such as pregnant women.
